# Supplementary material for: The Role of Dectin-1 Signaling in Altering Tumor Immune Microenvironment in the Context of Aging
Source: Front Oncol. 2021 Apr 22;11:669066. doi: 10.3389/fonc.2021.669066 (PMC8100664; doi:10.3389/fonc.2021.669066)
Supplement: Supplementary file 1 [file DataSheet_1.pdf]

Supplementary figures

Fig.S1

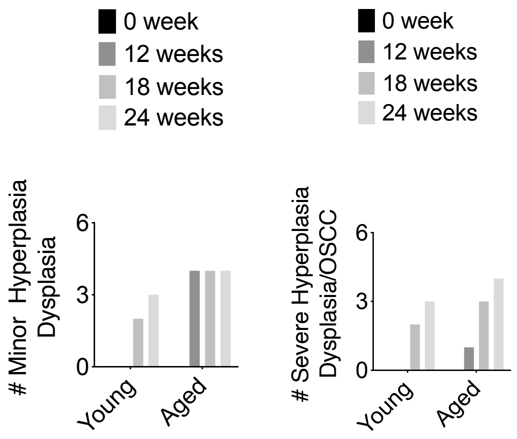

Fig.S1. Aged mice have earlier incidence of dysplasia and more severe progression of carcinogenesis compared to young mice. Mice were administered with 4-NQO as in Fig.1. Bar graphs showing the number of mice with hyperplasia/dysplasia in the group of 4 mice.

Fig.S2

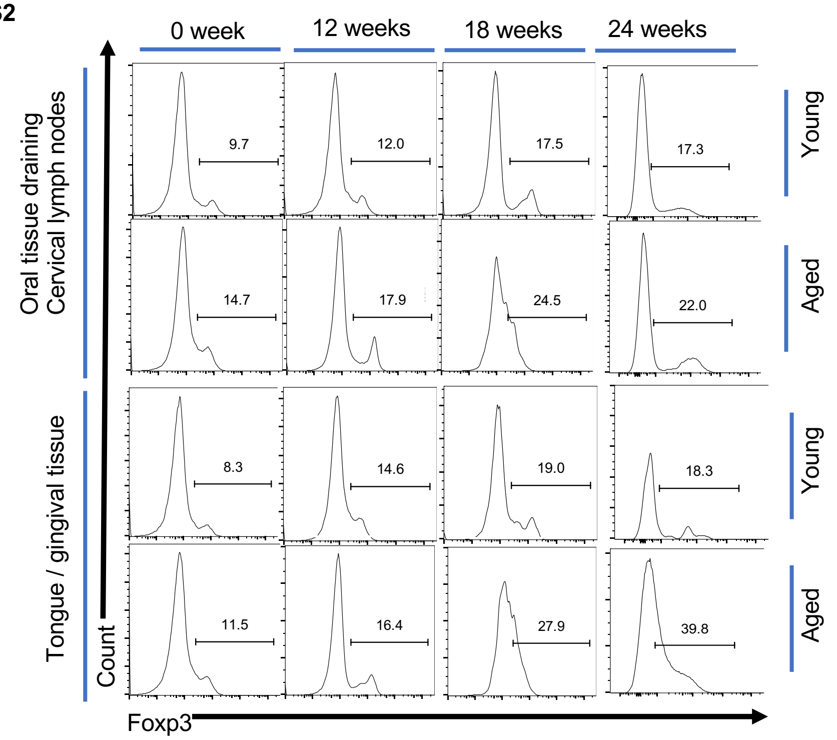

Fig.S2. 4-NQO treatment increases the proportions of $CD4^+T_{regs}$ during early stages of carcinogenesis in mice. Aged mice have higher proportions of $CD4^+T_{regs}$ compared to young mice, before and after treatment. 4-NQO was administered in drinking water (50 ug/ml) to mice (n=9/young or aged group) for 0,12, or 18 weeks. Samples were processed for flow cytometry. Contour plots gated on $CD3^+CD4^+$ lymphocyte singlets after dead cell exclusion.

Fig.S3

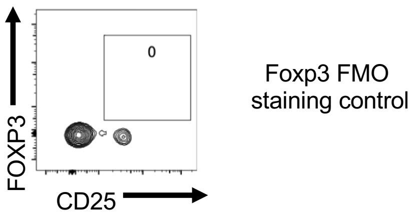

Fig. S3. FOXP3 FMO staining. Human oral tissue samples were obtained, processed for flow cytometry and stained with all the antibodies in the panel except FOXP3. Contour plots gated on $CD3^+CD4^+$ lymphocyte singlet cells after dead cell exclusion.

Fig.S4

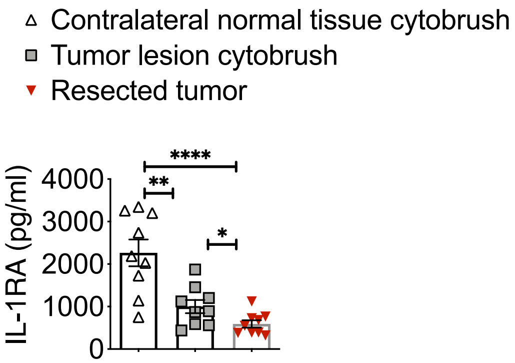

Fig.S4. IL-1RA levels are diminished in OSCC tumors. Human oral tissue samples were obtained either by cytobrushing or by excision under an approved IRB protocol. The single cell suspensions were restimulated with PMA/Ionomycin for 4 hours and cell supernatants were used for IL-1RA ELISA.

Fig.S5

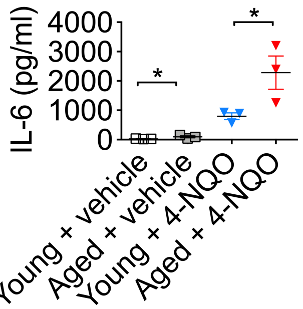

Fig.S5. Aged mice have significantly elevated levels of IL-6 in early stages of carcinogenesis compared to young mice. Mice were administered with 4-NQO for 12 weeks and tongue cells were re-stimulated with PMA/Ionomycin for 4 hours before supernatants from these cultures were used for IL-6 ELISA.

Fig.S6

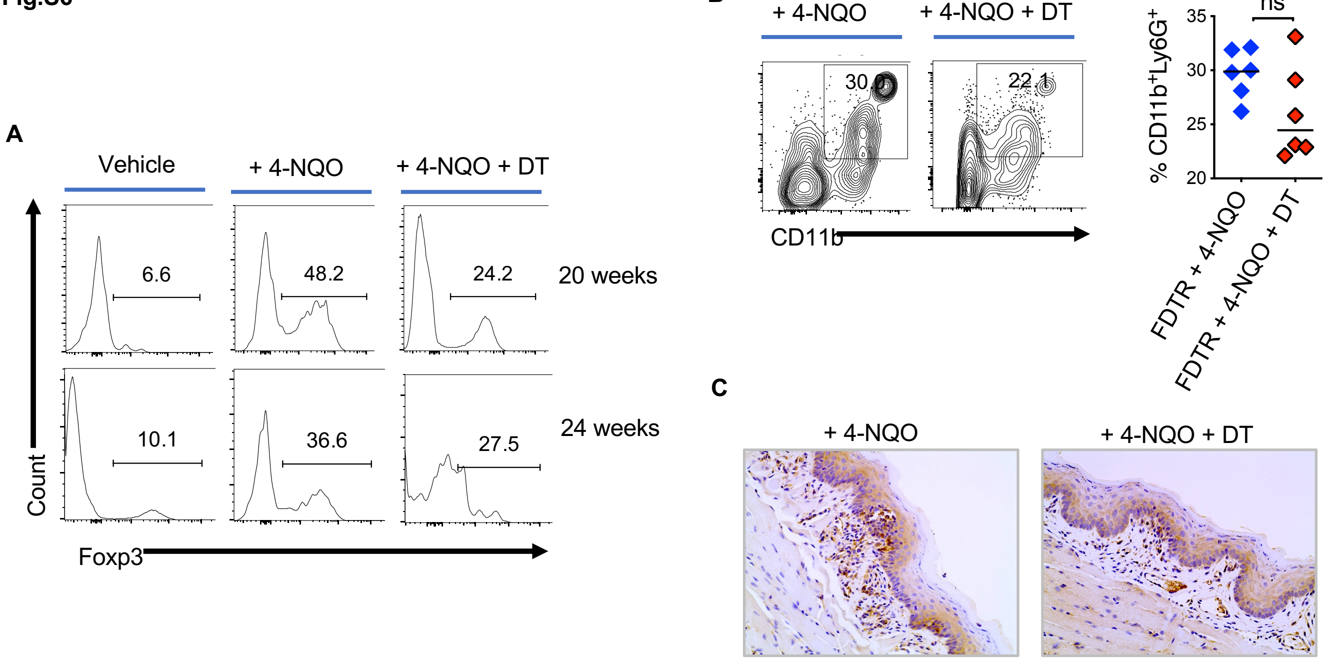

Fig.S6. Partial T_{reg} depletion does not affect IL-1β and MDSC proportions. T_{regs} were depleted in FDTR mice by injecting diphtheria toxin (DT) every 5 days between 16th -21st weeks of 4-NQO treatment. Flow cytometry contour plots showing $CD4^{+}Foxp3^{+}$cells (**A**) and MDSC (**B**, left). Statistical analysis of MDSC proportions (18 weeks) (**B**, right). **C**) IL-1β immunohistochemistry staining and microscopy were performed at 18 weeks of 4-NQO administration (400X magnification).

Fig.S7

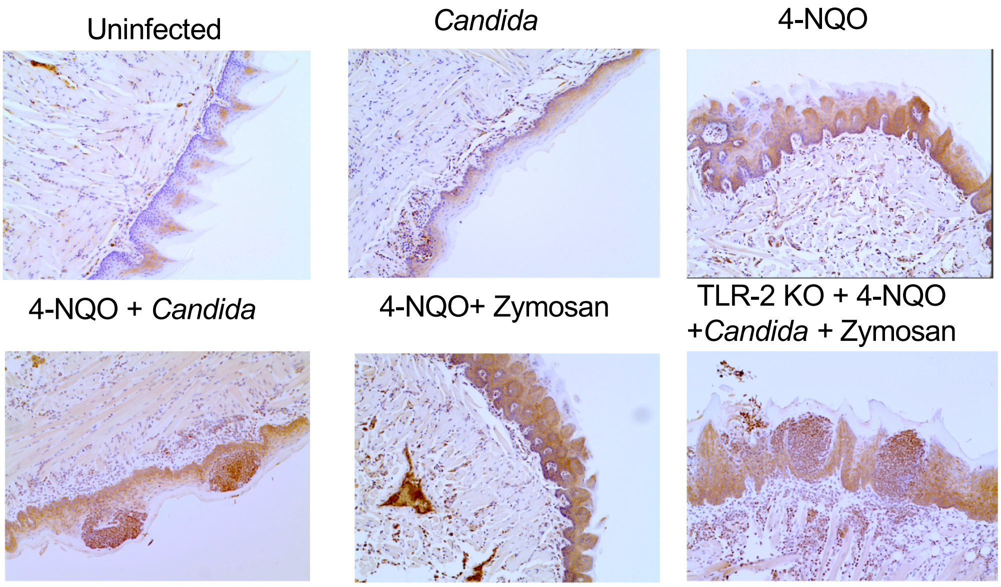

Fig.S7. *Candida* and Zymosan increase IL-1β expression in tongue during 4-NQO induced carcinogenesis. 4-NQO was administered in WT or TLR-2 KO C57BL/6 mice (6 mice/group). Zymosan (1mg) or *Candida* (10^7 blastospores) were applied sublingually under anesthesia every week between 4th and 8th weeks of 4-NQO administration. IL-1β immunohistochemistry staining and microscopy were performed at 12 weeks (200X magnification).

Fig.S8

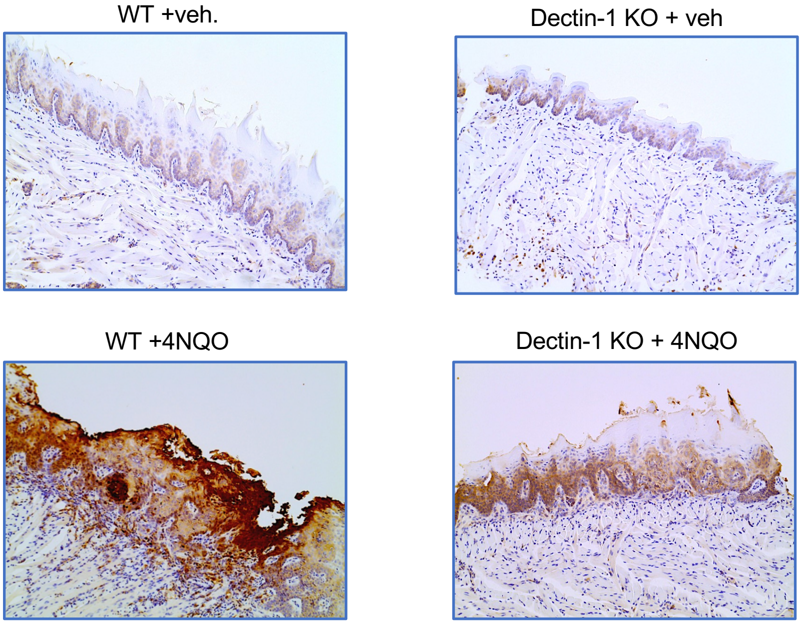

Fig.S8. Dectin-1 deficiency reduces IL-1β expression in tongue during 4-NQO induced carcinogenesis. Tongue tissues were processed for immunohistochemistry (IHC) and flow cytometry at 23 weeks after 4-NQO administration (200X magnification).

Fig.S9

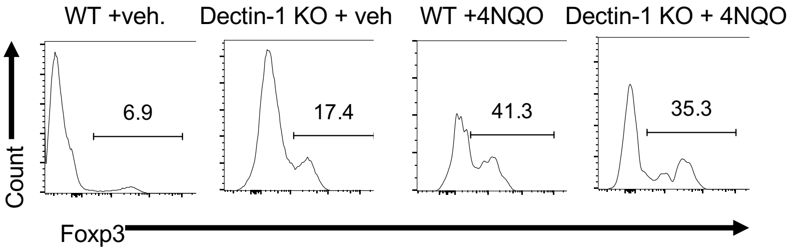

Fig.S9. 4-NQO carcinogenesis induced T_{reg} infiltration in tongue is significantly reduced with loss of Dectin-1. Flow cytometry contour plots showing $CD4^{+}Foxp3^{+}$cells, 23 weeks after 4-NQO administration.
